# Effect of Thermomechanical Treatments on the Phases, Microstructure, Microhardness and Young’s Modulus of Ti-25Ta-Zr Alloys

**DOI:** 10.3390/ma12193210

**Published:** 2019-09-30

**Authors:** Pedro Akira Bazaglia Kuroda, Fernanda de Freitas Quadros, Raul Oliveira de Araújo, Conrado Ramos Moreira Afonso, Carlos Roberto Grandini

**Affiliations:** 1 Laboratório de Anelasticidade e Biomateriais, Faculdade de Ciências (FC), Campus de Bauru, Universidade Estadual Paulista (UNESP), Bauru, 17.033-360 SP, Brazil; pedro.kuroda@unesp.br (P.A.B.K.); ff.quadros@unesp.br (F.d.F.Q.); 2 IBTN/BR – Institute of Biomaterials, Tribocorrosion and Nanomedicine - Brazilian Branch, Bauru, 17.033-360 SP, Brazil; raul.araujo@ifsp.edu.br; 3 Ciência e Tecnologia de São Paulo, Instituto Federal de Educação (IFSP), Barretos, 14781-502 SP, Brazil; 4 Department of Materials Engineering (DEMa), Federal University of Sao Carlos (UFSCar), São Carlos, 13565-905 SP, Brazil; conrado@ufscar.br

**Keywords:** titanium alloys, biomaterial, heat treatment

## Abstract

Titanium and its alloys currently are used as implants, possessing excellent mechanical properties (more suited than stainless steel and Co-Cr alloys), good corrosion resistance and good biocompatibility. The titanium alloy used for most biomedical applications is Ti-6Al-4V, however, studies showed that vanadium and aluminum cause allergic reactions in human tissues and neurological disorders. New titanium alloys without the presence of these elements are being studied. The objective of this study was to analyze the influence of thermomechanical treatments, such as hot-rolling, annealing and solution treatment in the structure, microstructure and mechanical properties of the Ti-25Ta-Zr ternary alloy system. The structural and microstructural analyses were performed using X-ray diffraction, as well as optical, scanning and transmission electron microscopy. The mechanical properties were analyzed using microhardness and Young’s modulus measurements. The results showed that the structure of the materials and the mechanical properties are influenced by the different thermal treatments: rapid cooling treatments (hot-rolling and solubilization) induced the formation of α” and β phases, while the treatments with slow cooling (annealing) induced the formation of martensite phases. Alloys in the hot-rolled and solubilized conditions have better mechanical properties results, such as low elastic modulus, due to retention of the β phase in these alloys.

## 1. Introduction

The first alloy developed specifically for use in humans is known as “vanadium steel (1900)”, a biomaterial produced for repairing bone fractures and for fixation screws used in orthopedics [1]. Currently, elements such as chromium, tantalum, zirconium, cobalt, titanium, niobium, tungsten, iron and nickel are also used as biomaterials, although the human body tolerates these elements only in small quantities [2]. Materials developed for use as biomaterials must possess certain favorable physical and chemical characteristics and must not induce inflammation, toxic reactions or allergenic symptoms in the host. They must be biocompatible, biofunctional, bioactive, bioinert and sterilizable [3].

Unfortunately, metal implants have been known to fail after prolonged use due to their high elasticity modulus compared to bone, and to their poor resistance to wear and corrosion in body fluids [4,5]. The bonding force between atoms determines Young’s modulus. This bond strength is related to the crystalline structure of the material and to the distances between atoms [6,7]. It is possible to change the bond strength between atoms by thermomechanical treatment [8]. Plastic deformations are another treatment that can alter the structure of titanium, thus changing the atomic bond strength and Young’s modulus [9]. The ω phase of titanium is known to have the highest modulus of elasticity, followed by the α and the α” phases; the β phase has the lowest modulus, due to its low atomic bond strength [10]. New alloys are being developed and investigated whose microstructure and mechanical properties can be altered with the addition of various elements, usually β-stabilizers [11,12,13,14,15,16].

Ti-Ta-Zr alloys are being developed for biomedical applications. Zhou et al. [17] indicate that the most promising of the Ti-Ta binary system alloys for use as a biomaterial is Ti-25Ta. Some authors assert that zirconium is a neutral element in titanium alloys, that is, its addition does not modify the structure of the material [18]. Recently, new research has shown that zirconium helps stabilize the β phase in α + β-type alloys, acting as a β-stabilizer [19,20,21,22,23,24]. Therefore, alloys of the Ti-25Ta-Zr system may be more promising as biomaterials, hence the addition of zirconium facilitates the β phase stabilization, in reason of the fact that β-type alloys tend to have a lower modulus of elasticity than alpha-type alloys. Besides aiding in the stabilization of the β phase, zirconium contributes to cost savings in the manufacture and commercialization of these alloys [25], because tantalum has a high market value and a high melting point, making production in high ladder more difficult.

The literature does not have great information about the structure, microstructure, phases and mechanical properties of Ti-Ta-Zr alloys. Yan at al [26] developed Ti-15Ta-xZr system alloys (x = 1.5, 5.5, 10.5, and 15.5 wt.%) manufactured by selective laser melting. Structural and microstructural analyses show that the alloys have the α and β phases and excellent elastic modulus values, highlighting the Ti-15Ta-10.5Zr alloy with a value of approximately 43 GPa. Biesiekierski et al. [27] developed new Ti-Ta-Zr system alloys with high tantalum and zirconium contents (Ti-45Zr-10Ta, Ti40Zr-14Ta, Ti-35Zr-18Ta, and Ti-30Zr -22Ta). The results showed that the alloys produced are of β-type and have elastic modulus like commercially pure titanium and Ti-6Al-4V (109-130 GPa). All alloys have good biocompatibility. 

Ti-25Ta-10Zr alloy is the only alloy of type α’+ α” + β reported in the literature. Hardness values are above commercially pure titanium, being hardening by the solid solution. Cytotoxicity assays showed that the alloy does not have a cytotoxic character; on the contrary, the alloy showed perfect cell division, maintaining morphology, indicating good integration between material and cell [28].

The objective of this work was to analyze the influence of thermomechanical treatments, such as hot-rolling and annealing heat treatments, on the structure, microstructure, microhardness and elastic modulus of ternary alloys of the Ti-25Ta-Zr system, with zirconium content varying from 0 to 40% in weight.

## 2. Materials and Methods 

The alloys were melted in an arc-melting furnace [22,28,29,30,31,32]. After melting, the ingots were submitted to a mechanical hot-rolling process at 1273 K, followed by air-cooling. 

Because the microstructure of the metal undergoes changes during rolling, an annealing heat treatment was used to reduce the internal stress caused by the mechanical rolling process. The purpose of the annealing treatment was to eliminate residual internal stress and most of the imperfections present in the material and to promote the growth of the grains [33]. 

Two types of heat treatments were performed. After thermomechanical processing, the samples were submitted to recrystallization heat treatments at 1273 K, remaining at this temperature for 21.6 Ks, followed by slow cooling (SC) (−5 K/min), and another similar treatment was followed by water quenching (RC) (~273 K). 

For structural analysis, X-ray diffraction (XRD) analysis (Rigaku model D/Max-2100PC) was used to obtain the diffractograms adopting the powder method, with Cu-Kα radiation, 20 mA current, potential 40 kV, for 3.2 s, range from 20° to 100°, and step of 0.02°, in the fixed time mode. 

Before microstructural analysis, samples were submitted to a standard metallographic preparation. Images were obtained using an optical microscope (Olympus model BX51M), a scanning electron microscope (SEM) (Carl Zeiss model EVO-015) and a transmission electron microscope (TEM) (FEI Tecnai G^2^ F20). TEM analysis was performed using a FEI Tecnai G2 F20 200 kV microscope with energy-dispersive X-ray spectroscopy (EDS).

Hardness measurements were performed in a microhardness tester (Shimadzu, model HMV-2). In each sample, 20 indentations were made with a 25-g load for 60 s [34]. For dynamic elastic modulus, E (GPa) measurements, the impulse excitation technique (ATCP, Sonelastic^®^) was used [35].

## 3. Results

### 3.1. Phase Identification

**Figure 1:**
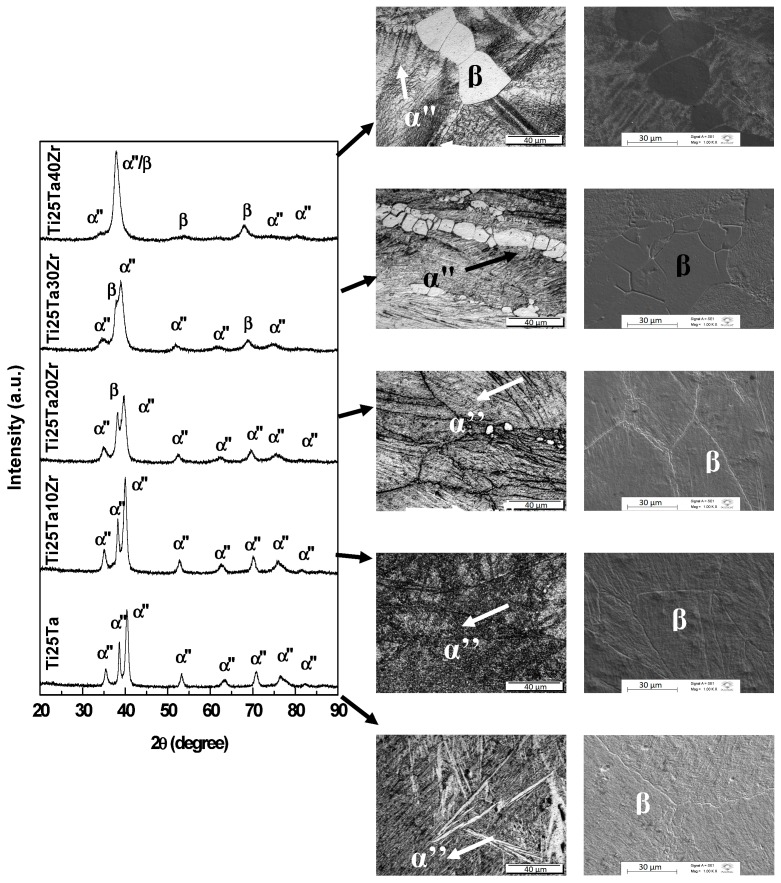
X-ray diffraction patterns and optical and SEM micrographs for Ti-25Ta-xZr system alloys after hot-rolling.

**Figure 2:**
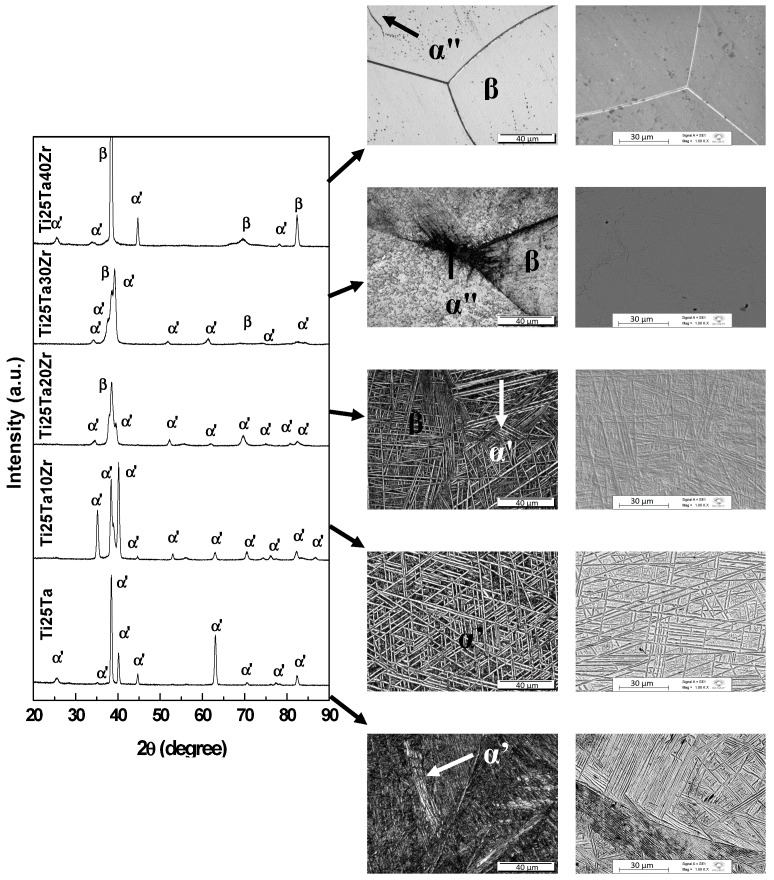
X-ray diffraction patterns and optical and SEM micrographs for Ti-25Ta-xZr system alloys after annealing heat treatment with slow cooling.

**Figure 3:**
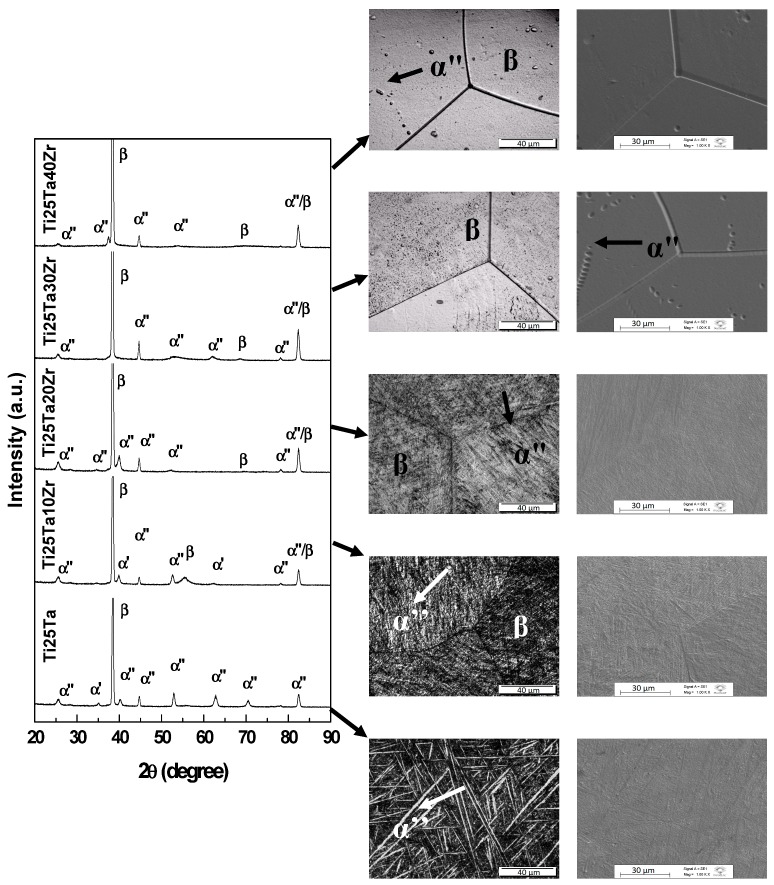
X-ray diffraction patterns and optical and SEM micrographs for Ti-25Ta-xZr system alloys after annealing heat treatment with rapid cooling.

The X-ray diffractograms and optical and SEM micrographs for Ti-25Ta-Zr system alloys after hot-rolling are shown in Figure 1; after annealing and with slow cooling in Figure 2; and in Figure 3, the results for samples subjected to annealing with fast cooling, are presented. As shown, in system alloys Ti-25Ta-Zr the crystalline structure was sensitive to thermomechanical treatments.

### 3.2. Mechanical Characterization

**Figure 4:**
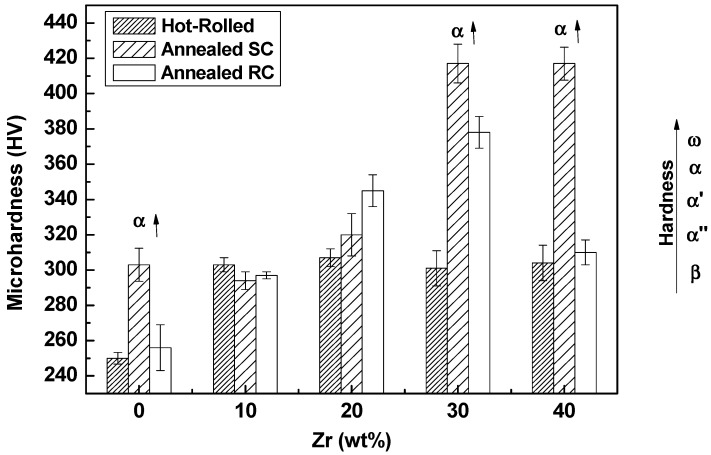
Vickers microhardness values of Ti-25Ta-Zr system alloys after hot-rolling and heat treatments, annealing with slow (SC) and rapid cooling (RC).

Figure 4 shows the hardness values for Ti-25Ta-Zr system alloys, performed in the samples after each processing condition included in this study. An anomaly for the hardness of Ti-25Ta-30Zr alloy can be observed after annealing with rapid cooling. This anomaly can be associated with the retention of phase [36,37].

**Figure 5:**
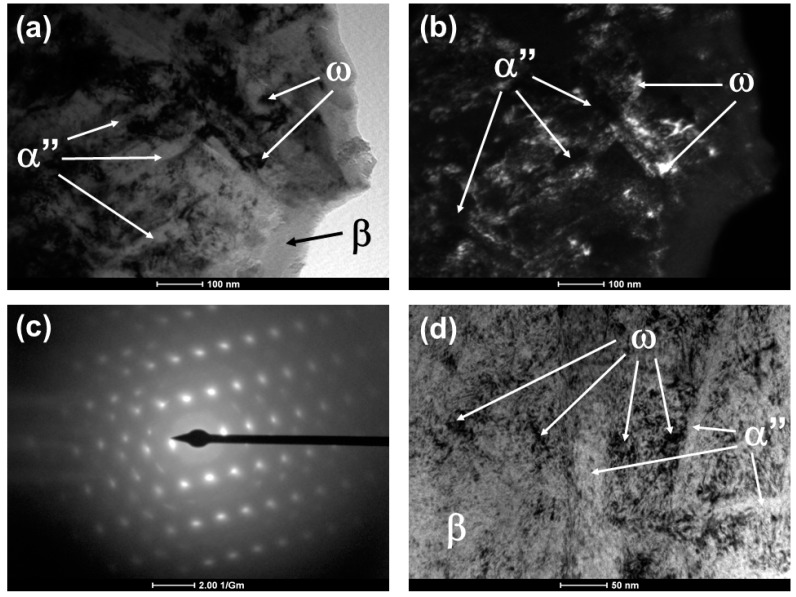
TEM images for Ti-25Ta-30Zr alloy after annealing with rapid cooling: bright-field (**a**), dark-field (**b**), diffraction of the selected area (**c**) and high-resolution image (**d**).

To verify the presence of ω phase, TEM measurements were made in the Ti-25Ta-30Zr alloy after annealing with rapid cooling, and the results, presented in Figure 5, show the bright (part (a)) and dark (part (b)) field images, the diffraction pattern (part (c)) and a high-resolution image of the selected area (part (d)). The presence of ω phase can be clearly observed [33].

**Figure 6:**
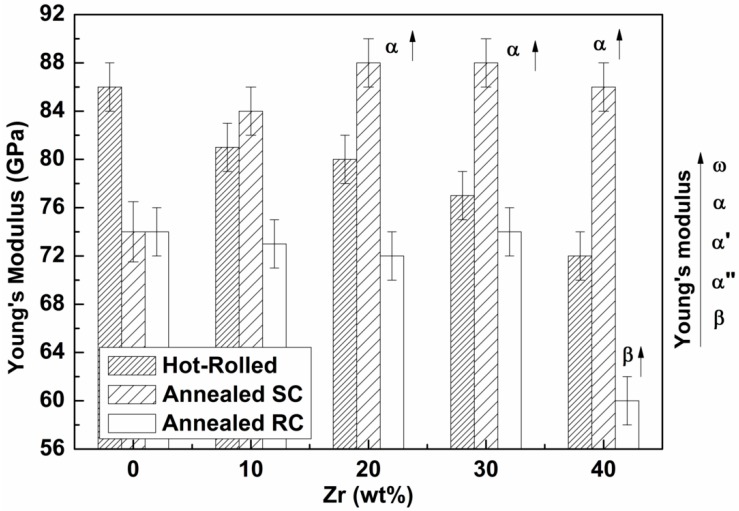
Young’s modulus for Ti-25Ta-Zr system alloys, in all studied conditions.

Figure 6 shows the values of Young’s modulus for Ti-25Ta-Zr system alloys, under all conditions investigated in this study. It can be observed that the values of elastic modulus decrease with the zirconium amount for the hot-rolled and annealed with rapid cooling samples. For the annealed with slow cooling, an increase of elastic modulus with the zirconium amount was observed. 

## 4. Discussion

It can be observed, in Figure 1, that Ti-25Ta and Ti-25aTa-10Zr alloys show only peaks of α” phase in the hot-rolled condition. For the Ti-25Ta-20Zr alloy, the diffractograms show the coexistence of peaks associated with α” and β phases, but those of β phase have lower intensity. Ti-25Ta-30Zr and Ti-25Ta-40Zr alloys have peaks that are associated with α” and β phases, too. It is evident that the zirconium acted as a β-stabilizing element, hence its presence increases the β phase precipitation.

After the annealing heat treatment with slow cooling, the orthorhombic α” phase changed to the hexagonal close-packed structure, α phase, and the β phase remained in the structure of the material. It can be clearly observed that heat treatment modified the phases of the alloys. Since the annealing was carried out at a temperature above β-transus and cooled slowly, it was provided the necessary thermodynamic conditions for the stabilization of the phases at high temperatures, if they change to stable phases at room temperature, in the case α”→α [38].

The alloys submitted to the annealing treatment with rapid cooling presented the metastable orthorhombic α” phase. In addition, alloys with a zirconium content above 20 wt% showed a more intense β phase peak at θ ~ 68°, indicating higher fraction and greater stabilization of bcc structure. It is stated in the literature that rapid cooling of titanium alloys from the β field can induce the formation of the metastable α” martensite since the fast cooling hinders the atomic rearrangement (retaining the β phase) and produces distortions in the crystalline structure [33]. In addition to detection of α” and β phases, in alloys with 0, 10, 20 and 30 weight percent zirconium, an ω phase peak can be visualized at θ ~ 78°. In the Ti-25Ta-40Zr alloy it was not possible to observe ω phase peaks, showing that zirconium acted as an omega phase suppressor.

Combining the results of XRD, a typical martensitic structure can be seen in Figure 1 to Figure 3, α” phase, and grains deformed due to plastic deformation in the Ti-25Ta, Ti-25Ta-10Zr and Ti-25Ta-20Zr alloys, in the condition after hot-rolling. After the annealing, there was recrystallization of the material leading to homogenization of the structure and grain size. In these alloys, after recrystallization with slow cooling, an α-type lamellar structure can be seen, with needles emerging from the grain boundaries and thinner needles parallel to the thick needles. In the annealed condition with rapid cooling, there was an increase in grain size, but the martensites phases are more refined, that is, the intra-grain needles are finer with this type of treatment. The images indicate that the cooling rate influences the microstructure in the material—slow cooling induces the formation of the α phase, and rapid cooling promotes the formation of the orthorhombic α” phase.

In Ti-25Ta-30Zr alloy after hot-rolling, the α” and β phases coexist; the micrographs exhibit an equiaxial structure of very fine needles distributed in some β-type distorted grains. After annealing with slow cooling, there was an increase in grain size and the α phase needles were within the grain boundaries. In annealing with water cooling, grains of the β-matrix can be observed, with small α” phase precipitates.

In the alloy with 40 wt% of zirconium, it is possible to observe small grains of the material with morphology characteristic of the β phase, in the after hot-rolling condition. The annealing treatments promoted the growth of these grains. Typical structures of the martensites are difficult to see in micrographs, although the x-ray diffractograms showed peaks of the α ’and α” phases.

A major problem in the manufacture of titanium prostheses is the mechanical conformation. A biomedical material must be easily shaped to facilitate its handling to produce screws, plates, and other shapes. The microhardness of a material indicates whether it conforms easily (low hardness value) or is a hard, brittle material. Further, biocompatible alloys must have a hardness appropriate to their specific application, because if they have very high hardness in relation to the implanted tissue, it can result in rapid tissue wear. Thus, many studies evaluate the microhardness in titanium alloys with respect to heat treatments, processing and the microstructure [39,40,41,42,43].

Figure 4 shows the hardness values for each process performed in the samples of Ti-25Ta-Zr alloys included in this study. According to the literature, among the phases formed in titanium alloys, the ω phase presents the greatest hardness value, followed by the α, α’, α” and β [44,45,46]. Other studies report that β-type alloys may have a greater hardness level than mastensitic phases α’ and α’’, because they have a stronger solid solution due to phase stability (ω > α’ > α” > β > α) [47].

As can be observed, after the hot-rolling process the alloys have an equal or lower hardness value, compared to the values of annealed alloys with slow cooling. From the X-ray diffractograms, it is possible to visualize the effect of hot-rolling, heat treatment and zirconium substitution in the structure of the material. After the hot-rolling, there is a formation of the α” phase for the alloys with zirconium up to 20%, and in those above 30% of zirconium, the β phase coexists with the α” phase. The annealing alleviated the internal stress from the mechanical process and modified the α” to the α phase in the alloys undergoing slow cooling. As mentioned above, α type titanium alloys have greater hardness compared to α” alloys. Consequently, the alloys in the hot-rolled condition tend to have a lower hardness value compared to the annealed alloys with a slow cooling rate [47].

Ti-25Ta-30Zr and Ti-25Ta-40Zr alloys annealed with slow cooling have high hardness values (~425 HV). This increase can also be explained by the presence of the α and β phases, which may be an obstacle to the dislocations motion.

From hardness measurements, it can be seen that for the lower (SC) cooling rate (5 K/s) condition, there is the formation of β + α phases for all the compositions. For water quenched (RC) condition after heat treatment, it might form precipitates of athermal ω phase due to higher hardness values (from 10 to 30%Zr) reached, but in a lower fraction than SC samples. Rapid cooling condition (RC) resulted in a greater stabilization of β phase for higher zirconium content (40%Zr), suppressing also the ω phase and decreasing the hardness value.

It was observed that the hardness of the hot-rolled alloys and the alloys subjected to the annealing treatment with fast cooling have similar values. The alloys in these two conditions have the same crystalline structure (α” and β), and therefore their mechanical characteristics are similar. Only in the Ti-25Ta-30Zr alloy (with α” and β phases), there is an “anomaly” with reference to the hardness value. This high hardness value can be an indication of the formation and highest precipitation of the ω phase in this alloy, as the fast cooling can retain the ω phase, which has the characteristic of hardening and weakening the material.

Due to the high hardness of the Ti-25Ta-30Zr alloy subjected to rapid cooling, a more detailed analysis of the microstructure of the material was performed to see the ω phase as the enhancer. Figure 5 shows the bright and dark field images of TEM, a bright field (BF) image and the selected area diffraction pattern (SAD) showing fine martensite α” phase needles with a width of approximately 50 nm dispersed in a β phase (bcc) matrix. Nanoscale precipitates of athermal ω phase from 5 to 20 nm are visualized in the TEM micrographs; their nucleation and precipitation are located close to the α” phase structures. Such athermal ω phase formation occurs in α + β type alloys and when such alloys are subjected to rapid cooling from high temperatures. The omega phase recipients are located intra-grain in the titanium structure, in circular format, visualized in nanometer scale, according to similar results of Niinomi (2016) and Homma (2018) [48,49]. Due to the precipitation of ω phase, Ti-25Ta-30Zr alloy submitted to rapid cooling showed a high value of hardness, as indicated in Figure 4 [50,51].

Figure 6 shows the values of the dynamic elastic modulus of the samples of all Ti-25Ta-Zr alloys used in this study, under all investigated conditions. In this figure, it can be observed that Young’s modulus of the alloys studied after hot-rolled condition was approximately 86 GPa for the non-zirconium alloy and reached a minimum value of 72 GPa for the alloy with the highest zirconium content (40 wt%). After hot-rolling, there is an increase in the percentage of the β phase, which decreases the elastic modulus, since the β phase has the smallest modulus value among the phases in titanium alloys. The hot-rolling process generates a high cooling rate when the sample is passed through the mill roller, retaining the β phase.

Annealing with slow cooling, by contrast, increased the value of Young’s modulus with the addition of zirconium. The treatment decreased the percentage of the β phase and modified the α” to α phase due to the recrystallization of the sample. The alloys with the α phase have higher elastic modulus values than α” alloys. Annealing with rapid cooling decreased the modulus of these alloys, as this type of treatment at high temperatures and rapid cooling induces the formation of the β phase, which has the lowest modulus value in titanium alloys. The Ti-25Ta-40Zr alloy has the lowest modulus of the studied alloys of E = 60 GPa.

It can be noted by the hot-rolled condition, that increasing zirconium fraction leads to the decreasing of the elastic modulus from 86 down to 72 (GPa), due to a combination of stress-induced martensite formation and β phase stabilization with suppression of ω formation. Evaluating the elastic modulus E (GPa) for a lower cooling rate leads to a tendency like the hardness measurements, where the appearance of the α phase in high zirconium alloys leads to an increase in mechanical property values, increasing the modulus from 74 to 88 GPa. Compared with the rapid cooling condition, the modulus only just decreased significantly from 74 down to around 60 GPa for higher zirconium content (40%), due to greater stabilization of β phase. 

The Ti-25Ta-xZr alloys produced in the annealed condition with fast cooling have a better modulus compared to Ti-cp and other commercial biomedical metals, such as Ti-6Al-4V, 316L and Co-Cr [12]. Among the produced alloys in this study, the Ti-25Ta-40Zr has the lowest elastic modulus value (60 ± 2) GPa, which represents twice the modulus of elasticity of human cortical bone [52].

Although the Ti25Ta-40Zr alloy is promising in the biomedical field, new mechanical property testing and corrosion analysis are still required, as the surface of metals in contact with body fluids may corrode, reducing implant longevity, and to perform biological biocompatibility to verify if the material has cytotoxic character in the human organism.

## 5. Conclusions

The results detailed above lead to the following conclusions.

- X-ray diffraction and microscopy reveal that the crystalline structure of the alloys was sensitive to the addition of zirconium and sensitive to thermomechanical treatments.
- The zirconium acted as a β-stabilizing element combined with tantalum in Ti-25Ta-xZr alloys.
- Alloys subjected to a slow-cooling annealing heat treatment have the α phase in the crystalline structure.
- The alloys subjected to a fast-cooling annealing heat treatment have the α” phase, can retain the β phase with high zirconium content and rapid cooling precipitates the omega phase, but high levels of zirconium suppress this phase.
- Hardness increased due to solid solution hardening and due to ω phase and α-phase precipitation in alloys subjected to slow-cooling annealing heat treatment.
- The Young’s modulus decreases with high levels of zirconium due to the stabilization of the β phase.
- The thermomechanical process and heat treatment with rapid cooling retain the β phase, improving mechanical biocompatibility with the decreasing of the elastic modulus values of the materials down to E = 60 GPa for Ti-25Ta-40Zr alloy.
